# Intentional binding decreases during learning: Implications for sense of agency

**DOI:** 10.1177/17470218251349521

**Published:** 2025-06-02

**Authors:** Silvia Seghezzi, Elisabeth Parés-Pujolràs, Patrick Haggard

**Affiliations:** 1 Institute of Cognitive Neuroscience, University College London, London, UK; 2 School of Psychological Sciences, Birkbeck University of London, London, UK; 3 School of Electrical and Electronic Engineering and UCD Centre for Biomedical Engineering, University College Dublin, Dublin, Ireland

**Keywords:** Sense of agency, intentional binding, learning

## Abstract

The sense of agency refers to the subjective experience of controlling one’s own actions and their outcomes. While agency is often thought to increase with better performance, it remains unclear how it evolves during learning. In this study, we investigated how the sense of agency changes as individuals learn when to act through reinforcement-based adaptation. We used intentional binding (IB)—a widely used, though debated, proxy measure for agency-related processes—to track temporal compression between actions and outcomes during a time-based learning task. Across four experiments, we found that IB decreased with learning, but only when feedback was imprecise yet stable, and when the outcome used to probe IB was irrelevant to the learning task. These results suggest that agency-related processes, as indexed by IB, may diminish when adaptation guides action selection, and when the outcome becomes less epistemically relevant. We discuss the possible implications of these changes in IB with learning for the sense of agency.

## Introduction

Voluntary actions are accompanied by a subjective experience of controlling one’s own actions and their consequences in the external world. This feeling has been called the “sense of agency,” and it captures the experienced association between actions and outcomes (Haggard, 2017). The sense of agency is a fundamental aspect of our conscious experience of acting and is crucial for our ability to interact with the environment effectively.

Learning is the process of acquiring new knowledge, skills, or behaviors through experience. It can occur at different levels of complexity, from simple associative learning to more complex forms of skill acquisition like reinforcement-guided learning (Rescorla, 1972; Skinner, 1974; Sutton, 1988; Sutton & Barto, 2018; Thorndike, 1898). Learning enhances individuals’ ability to select their actions to achieve the desired goals. As individuals gain experience with a task or situation, they develop an internal model of which actions are most likely to generate the desirable outcomes and which are not. This model guides their action selection by biasing them toward actions that are more likely to be successful. In this way, learning what to do (and/or when to do it) allows agents to acquire *control* on their environment by selecting the most efficient action and timing to achieve the intended outcome.

In general, better performance seems to be associated with a stronger self-reported sense of agency (Metcalfe et al., 2013; van der Wel et al., 2012; Wen et al., 2015). For example, van der Wel et al. (2012) investigated how the sense of agency developed while people learned a haptic coordination task. They showed that better performance was associated with a stronger sense of control, measured through a self-report rating from 0 (*no control*) to 100 (*complete control*).

However, our sense of agency extends beyond explicit, self-reported, judgments. It has been argued that, in everyday life, the sense of agency is often experienced as a subtle, background feeling that is very different from an explicit propositional judgement (Haggard, 2017; Synofzik et al., 2008). Intentional binding (IB) refers to the perceived compression of time between a voluntary action and its outcome. First introduced by Haggard et al., (2002), IB has been used as both an experimental paradigm and an implicit proxy measure to study mental representation of action–outcome linkage. Early studies established this phenomenon by contrasting active and passive movements, revealing that people perceive the interval between an action and its outcome as shorter when they actively initiate the action. Conversely, when the outcome occurs independently of the person’s action—for example, when it follows a passive movement—the interval is often perceived as dilated, or at least as less compressed. This comparison suggested IB as proxy measure for agency-related processes (Grünbaum & Christensen, 2020; Haggard, 2017).

Several studies have reported a positive correlation between IB and explicit agency judgments, showing that the stronger a person’s explicit sense of having caused an outcome, the greater the temporal compression they experience (Galang et al., 2021; Imaizumi & Tanno, 2019; Pyasik et al., 2018). However, this relationship is not always observed, as other studies have found weak or inconsistent correlations between IB and explicit agency ratings (Schwarz et al., 2019; Siebertz & Jansen, 2022), no differences between active and passive conditions (Kirsch et al., 2019; Kong et al., 2024; Suzuki et al., 2019), or no relation to action autonomy (Antusch et al., 2021). These findings contribute to an ongoing debate regarding the nature of IB and whether it reliably reflects sense of agency or arises from alternative mechanisms, such as multisensory integration (Hoerl et al., 2020; Klaffehn et al., 2021), attention (Cao, 2024; Schwarz & Weller, 2023), or other methodological confounds (Gutzeit et al., 2023; Reddy, 2022). Some of the inconsistencies relate to differences in the experimental designs used. Binding and compression phenomena are certainly not unique to agency. In general, one might expect to find binding whenever any two events are associated (Hume, 1874). However, a specific, *additional* binding or perceptual compression effect may occur when a voluntary action is followed by an outcome, over and above the binding that may occur between a control event and the same outcome. In fact, the basic IB paradigm has been used in experimental designs to test many different factors, including outcome valance, free versus forced choice (Beck et al., 2017; Borhani et al., 2017; Caspar et al., 2016), outcome predictability (Moore & Haggard, 2008; Rashidi et al., 2021) causal attribution—including temporal contiguity (Ruess et al., 2017), causal beliefs (Desantis et al., 2011), and judgments of causality (Spaccasassi et al., 2022). Not all of these designs involve an involuntary control condition, so the qualification IB is not always evidenced. However, many of these studies involved contrasts between conditions which operationally define a factor that influences to temporal binding between action and outcome. The use of IB as a proxy measure for agency-related processes remains controversial. The effect has been shown by several studies to be sensitive to a number of factors that one might be associated with agency, notably intentional action (Engbert & Wohlschläger, 2007; Haggard & Clark, 2003; see also Wiesing & Zimmermann, 2024 for a recent replication attempt that did not reproduce the findings of Suzuki et al. (2019)). A meta-analysis in 2019 suggested that an effect of intentional action did indeed exist (Tanaka et al., 2019), alongside components of binding due to other factors, notably predictability.

Only one study has directly examined the relationship between learning and IB. Pansardi et al. (2020) compared expert pianists and nonmusicians to assess how long-term sensorimotor training influences IB. They found that lifelong exposure to action–outcome contingencies during musical training enhances IB, possibly due to stronger action–effect predictions. However, no study has yet investigated how the IB changes in a reinforcement learning setting within a short experimental session, where participants refine their motor actions based on probabilistic feedback. These two learning processes—lifelong skill acquisition and trial-and-error adaptation—depend on different mechanisms (Haith & Krakauer, 2013), and therefore might influence IB in different ways.

This study investigated the relationship between trial-and-error learning and the sense of agency, focusing on the timing of actions and using IB as a proxy measure for agency-related processes. We used a temporal reinforcement learning task to study voluntary action (Travers et al., 2021). While prior research on learning has primarily explored what action to take based on the value of different alternatives (Di Costa et al., 2017; Majchrowicz et al., 2020), no study has specifically examined learning when to act and its impact on the IB. Temporal decision-making offers the advantage of providing a continuous measure of learning, rather than discrete action choices.

However, the relationship between learning and sense of agency is not always straightforward. Recent studies suggest that the sense of agency is influenced not only by an individual’s internal model of action but also by multiple additional factors (Christensen et al., 2016; Moore & Haggard, 2008; Moore et al., 2009). Therefore, in our study, in addition to the effect of learning, we aimed to examine how feedback structure influences learning and IB. In one condition, feedback was highly precise but making it more difficult to obtain and maintain stable positive reinforcement. In the other condition, feedback was less precise but more stable, providing less informative guidance for action selection but making positive reinforcement easier to achieve and maintain over time. In sum, we aimed to examine how trial-and-error adaptation interacts with feedback structure to shape the IB over time.

## Experiment 1

### Materials and methods

#### Participants

Participants were recruited using Prolific (www.prolific.co). We included participants aged between 18 and 35, fluent in English, right-handed, and with no history of any mental health or neurological condition.

A power analysis was conducted to determine the required sample size to achieve a desired level of statistical power. Assuming an alpha level of .05 and a desired power of 0.80, an effect size of 0.50 was estimated based on the effect of “Experiment Phase,” F(1, 54) = 17.230, *p* < .01, partial η^2^ = .244 reported in van der Wel et al. (2012). The analysis revealed that a sample size of at least 13 participants would be needed to achieve the desired power level with the estimated effect size. However, since our study requires a regression approach rather than factorial one, a larger sample size may be necessary to achieve adequate statistical power and reduce the risk of Type II errors. Therefore, 26 participants (9 female, mean age ± SD: 22.9 ± 3.6 years) were included in this experiment. This power analysis was based on a different dependent variable (explicit ratings of control) than the one used in our study (IB). However, at the time the power analysis was conducted, no studies were available that examined the relationship between learning and IB, and van der Wel et al. (2012) provided the closest available benchmark. No participant was excluded from the analyses according to the participants’ screening procedure (see Supplemental Materials).

#### Procedure

The experiment was approved by the UCL ICN ethics committee (ICN-PH-PWB-22-11-18A). Once participants clicked on the survey link on Prolific, they reviewed and completed the consent form before being redirected to the platform for online experiments (PsyToolkit, see the next section for more details).

Before starting the experiment, participants underwent a brief sound check that consisted in reporting the correct number of sounds presented through headphones (3, 2, and 4, 500 Hz pure tones of 50 ms each, presented every 500 ms). If participants provided the correct sequence of sound numbers, they were redirected to the experiment; otherwise, they could repeat the test.

Participants received a £7.50—per hour reimbursement upon completing the experiment, plus a bonus depending on their performance.

#### Task

The experiment was programmed in PsyToolkit, version 3.4.0 (Stoet, 2010 2017).

The task consisted of a modified version of the temporal reinforcement learning paradigm developed by Travers et al. (2021).

**Figure 1.**
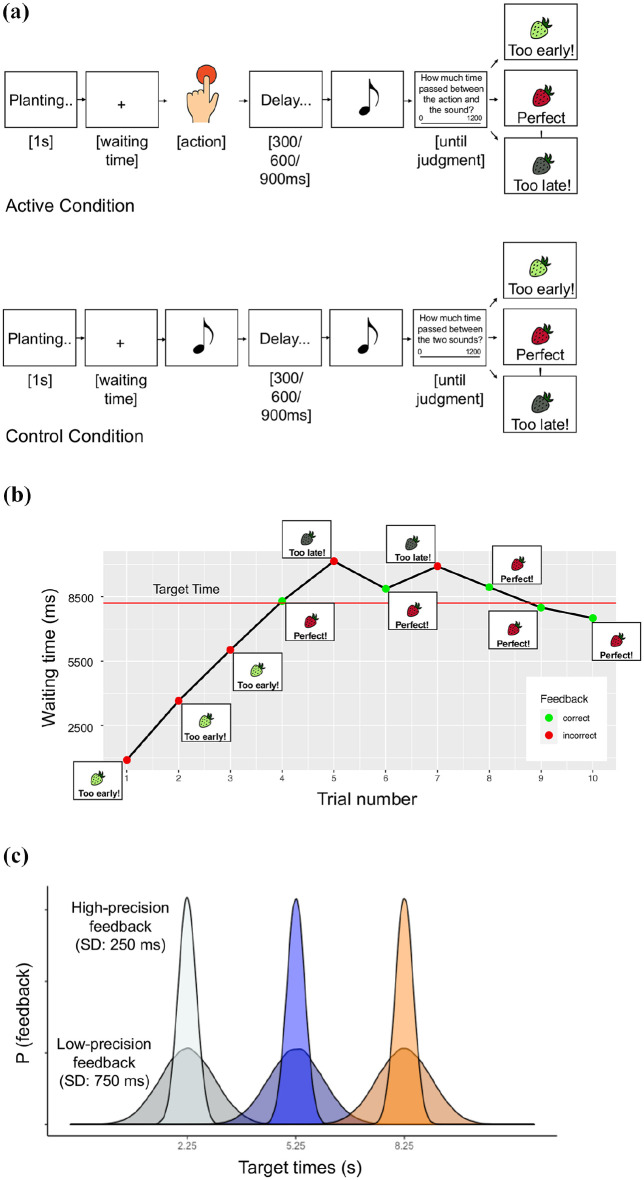
(a) *Temporal reinforcement learning task (Experiments 1 & 2)*. A seed was planted in the ground at the start of each trial, and participants had to wait an unknown time before pressing a button to harvest it. Feedback helped participants improve their performance. Before receiving feedback on the waiting time, they also had to estimate the delay between the time of their button press (or time of a first sound, in the control condition) and the time of the following sound. (b) *Block example with waiting times for a single participant (only 10 trials are shown)*. Horizontal bars show the block optimal target time. (c) *Feedback probability distribution under different conditions*. The precision of the feedback was manipulated by drawing from a probability distribution centered on the block’s target time, but with a standard deviation of either 250 or 750 ms. A standard deviation of 250 ms implied higher feedback precision but fewer positive feedbacks, while 750 ms corresponded to a lower feedback precision, and thus more positive feedbacks.

Participants were asked to learn, through experience, the best time to act. Participants acted as farmers, learning to harvest a seed planted at the beginning of each trial (see Figure 1a).

Each trial started with the seed being planted in the ground (“Planting. . .”). After 1 s, a fixation cross appeared in the center of the screen. After the cross appearance, participants could press the spacebar to “harvest” the fruit/vegetable. Participants did not know in advance the optimal waiting time to harvest the fruit/vegetable. Instead of a fixed threshold for determining correct responses, the feedback was probabilistic (see below for a more precise description), meaning that participants had to learn the probability of obtaining positive feedback rather than simply identifying a strict time cut-off. They had to discover this probability structure through trial and error.

Each block was associated with a different fruit/vegetable and an optimal waiting time (also called “target time” through the text), which participants must learn from signed error feedback. If participants harvested the fruit/vegetable at the right time, they gained a small extra cash bonus (£0.01). If they harvested the fruit/vegetable before it was ready or too late, they lost a small cash amount (£0.01). Importantly, losses functioned as a signed error signal, since the participants were told if their response was too early or too late. The “early” feedback showed the green fruit accompanied by the text “Too early!” and the “−0.01£” notice of loss. The “correct” message consisted of the picture of the ripe fruit/vegetable, accompanied by the text “Perfect!” and the “+0.01£” win notice. The “late” message consisted of the gray fruit/vegetable, accompanied by the text “Too late!” and the “−0.01£” notice of loss (see Figure 1b).

Participants learned to adjust their waiting time through feedback until they were reliably rewarded. The optimal strategy to complete the task was to initially explore the effect of acting after different delays, monitor the outcomes/feedback of each action, and then exploit the waiting time that was associated with the greatest probability of reward.

We introduced a control condition without voluntary actions. The control blocks were presented as “computer blocks.” Participants made no actions, but paid attention to when the computer “decided” to harvest the fruit. In each trial, after the fixation cross appeared at the center of the screen, participants heard a tone (500 Hz, 50 ms) corresponding to the time the computer harvested the fruit/vegetable. These tones were selected by shuffled replay from the participant’s own RTs in the active block. This control condition differed from other control conditions typically used in previous IB experiments involving passive movement execution (Haggard et al., 2002). However, here we were not interested in proving the existence of the intentional binding effect, but rather in seeing how binding for actions and tones varied with learning. The control condition was therefore used here to control for the generic effects of attention or expectation of events on time perception.

In the control condition without voluntary actions, participants were shown the feedback for the computer’s action, consisting, in fact, of the action feedback associated with the corresponding active block’s trial.

The precision of the feedback was manipulated, by drawing from a probability distribution centered on the block’s target time, but with a standard deviation of either 250 or 750 ms. A standard deviation of 250 ms implied higher feedback precision, while 750 ms corresponded to a lower feedback precision (see Figure 1c).

For each actual response time of the participant, a standardized (Z) score was computed as (RT—target)/standard deviation. The Z score served then as an input to extract the associated probability of having “correct” feedback according to a two-tailed hypothesis (to obtain the same probability for identical positive and negative distances from the target). The probability of a “correct” feedback for each possible Z score was determined beforehand and implemented as a set of 10% probability bands. Hence, to extract the *p*-value from a Z score in each given trial, the band containing the target Z value was identified, and the corresponding probability value was retrieved.

The probability of receiving negative feedback was computed as 1—the probability of “correct” feedback. If participants pressed the spacebar too early, they could probabilistically either receive an “early” or “correct” message depending on the probability distribution. If the participant pressed the spacebar after the optimal waiting time, they could probabilistically either receive a “correct” or “late” feedback depending on the probability distribution.

Low-precision feedback led to fewer errors, because it implied greater tolerance for keypress timing; therefore, participants received more frequent positive feedback in the 750 ms condition. However, this feedback would be less informative for guiding future action selection than in the condition with more precise feedback. In other words, even though participants made fewer errors in action timing when the standard deviation was 750 ms, they were less certain about the optimal waiting time to act.

To measure the IB effect (Haggard et al., 2002), participants’ actions were followed after a variable delay of 300/600/900 ms by an auditory tone (500 Hz, 50 ms). The tone was irrelevant to the primary task of making the action at the right time. Participants were asked to estimate the time interval between the action and tone by clicking with the mouse on the corresponding position of a continuous scale ranging from 0 to 1.2 s, and with markings at the endpoints (see Figure 1 for a graphical representation). The time estimation was provided before participants received feedback on their performance.

In the control condition without voluntary actions, participants estimated the time delay between the initial tone corresponding to the time the computer harvested the fruit/vegetable and a second tone presented after a variable delay of 300/600/900 ms (as in the active condition).

IB effects vary with outcome valence (Christensen et al., 2016; Takahata et al., 2012; Yoshie & Haggard, 2013). To avoid valence confounding learning effects, the feedback indicating whether the action was successful (i.e., positive or negative reinforcement) was presented only *after* participants had completed IB judgment. By temporally separating the perceptual timing judgment from the reinforcement feedback, we ensured that the IB measure was not directly influenced by the emotional or motivational salience of the outcome.

Before starting the experiment, participants completed one active block and one control block to get familiar with the task and check their understanding. In these training blocks, the optimal waiting time to be learned was 3 s, and the precision of the feedback probability distribution was 500 ms. The action–outcome delays were 200/500/1000 ms. In the first three trials, participants received feedback on their time estimation with notification of the actual action–outcome delay after the temporal judgment. Thereafter, feedback was as in the main experiment.

Participants completed 12 blocks in total, provided by the factorial combination of condition (Active/Control), feedback precision (250/750 ms), and optimal target time (2.25/5.25/8.25 s). The blocks were presented in a randomized order with the constraint that blocks with the same target time could not be consecutive, and the first block was always active. Each block consisted of 21 trials (7 trials at each action–outcome delay in randomized order).

#### Data analysis

All analyses were performed by means of the statistical software R (4.0.3) and the lme4 package (Bates et al., 2014).

Trials in which reaction times exceeded ±3 *SD* (0.5%) from the mean of the whole dataset were discarded from the analyses.

Variables were assessed for normality using histograms and Cullen and Frey graphs (Cullen & Frey, 1999). Variables showing substantial positive skewness were log-transformed prior to analysis to improve model fit (Osborne, 2002).

#### Performance

To measure participants’ performance, we computed the absolute difference between participants’ waiting time on each trial and the target waiting time for that block. Supplementary analyses on performance are reported in the supplementary materials.

#### Learning

In line with Travers et al. (2021), we computed the absolute change in participants’ waiting time from one trial to the next, |dWT|, as a trialwise measure of participants’ learning. While the signed error between actual and ideal interval performance measures performance on each trial, the unsigned |dWT| measure captures the magnitude of model update required from one trial to the next, and thus provides a good measure of learning. Thus, |dWT| lets us characterize each participant’s transition from an exploration phase where the time of action is varied strongly from one trial to the next (therefore, high |dWT|) an exploitation phase (essentially repeating time of action from the previous trial, therefore, low |dWT|).

Supplementary analyses on |dWT| are reported in the Supplemental Materials.

#### Intentional binding

The IB effect (Haggard et al., 2002) was computed as the difference between the estimated and the real duration of the action–outcome delay. Negative values represented an underestimation of the delay (i.e., a time compression), and positive values an overestimation. Stronger sense of agency has been associated with underestimation of the action–outcome delay (for a review see Moore & Obhi, 2012).

To assess the relation between IB and learning when to act, we used mixed-effects models to predict IB from our measure of updating |dWT|, condition (Active/Control), feedback precision (250/750 ms) and action–outcome delay (300/600/900 ms). Participant was modeled as random intercept. Learning, condition, feedback precision, and action–outcome delay were modeled as fixed effects.

Post hoc comparisons were performed on significant interaction effects by means of the packages *lsmeans* or *lstrends* and corrected according to the Tukey method. When needed, significant three-way interactions were also explored by means of separate analyses.

Data and scripts are available at the following link: https://osf.io/w7hs9/?view_only=df9ee62f3e0942c39c0ea8c57388ac19 .

### Results

#### Performance

**Figure 2.**
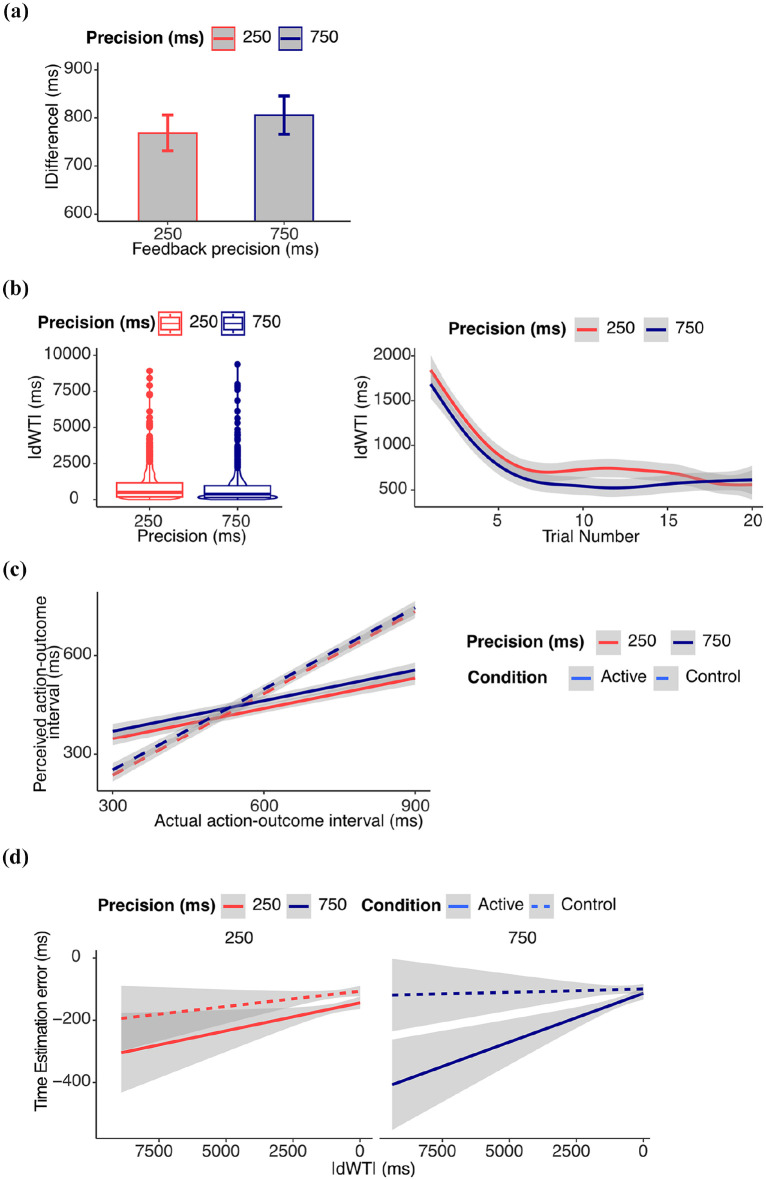
*Results from Experiment 1.* (a) *Performance*: absolute difference (ms) between waiting time and target waiting time. Figure shows the untransformed data, and error bars represent within-subject standard errors, computed using the standard error of paired differences. Statistical results refer to log-transformed data. (b) *Learning*: Learning progress measured as the absolute change in waiting time (|dWT|) from one trial to the next. Higher values indicate larger trial-to-trial adjustments (exploration), while lower values suggest more stable estimates (exploitation). Figure shows the untransformed data, statistical results refer to log-transformed data. (c) *IB*: estimated action–outcome interval. The perceived action–outcome intervals were shorter in the active than in the control condition. (d) *Relation between IB and learning*: time estimation error (difference between estimated and actual delay). In the active condition, the IB effect decreased with learning, as measured by |dWT|. This effect was statistically significant only in the 750 ms feedback precision condition.

Results from supplementary analyses on performance are reported in the Supplemental Materials and in Figure 2a.

#### Learning

Results from supplementary analyses on |dWT| are reported in the supplementary materials and in Figure 2b.

#### Intentional binding

Our core question was whether the IB would increase or decrease with learning when to act, and how this would be modulated by the precision of feedback information. We therefore focus here on the interaction between |dWT|, feedback precision, and active/control condition. All other results from the model exploration are reported in the Supplemental Materials.

We observed a significant three-way interaction between condition, |dWT|, and feedback precision (β = .017 (*SE* = 0.020), 95% CI [−0.023, 0.057], χ^2^(1) = 4.05, *p* = .044). Post hoc comparisons showed that the reduction of the IB effect with learning was greater in the active than control condition only in the 750 ms feedback precision condition (*p* = .002). To further confirm the results, we tested the significance of the slopes of the effect of |dWT| on IB separately for each condition (see Supplemental Materials). This was significantly greater than zero only in the active 750 feedback precision condition (β = −.021 (*SE* = 0.007), 95% CI [−0.035, −0.008], χ^2^(1) = 9.32, *p* = .002). The results are reported in Figure 2c and d.

## Experiment 2

Experiment 2 aimed to replicate the effect of IB reduction with learning in an independent sample of participants. We also changed the target waiting times that participants had to learn. This was done to ensure that the observed effects were not specific to a particular timing range and to rule out any confounding influence of absolute waiting time on learning or IB. By varying the target times across experiments, we aimed to demonstrate that the relationship between learning and IB generalizes across different temporal contexts.

### Materials and methods

#### Participants

An independent sample of 26 participants (7 female, mean age ± *SD*: 21.7 ± 3.6 years) took part in this experiment. Three participants were excluded from the analyses in compliance with the participants’ screening procedure (see Supplemental Materials).

#### Task

The task used in Experiment 2 exactly replicated those of Experiment 1 except for the waiting times to be learned (2.25/3.5s/4.75 s).

#### Data analysis

The analyses for Experiment 2 exactly replicated those of Experiment 1.

Trials in which reaction times exceeded ±3 *SD* (2.7%) were discarded from the analyses.

Data and scripts are available at the following link: https://osf.io/w7hs9/?view_only=df9ee62f3e0942c39c0ea8c57388ac19.

### Results

#### Performance

**Figure 3.**
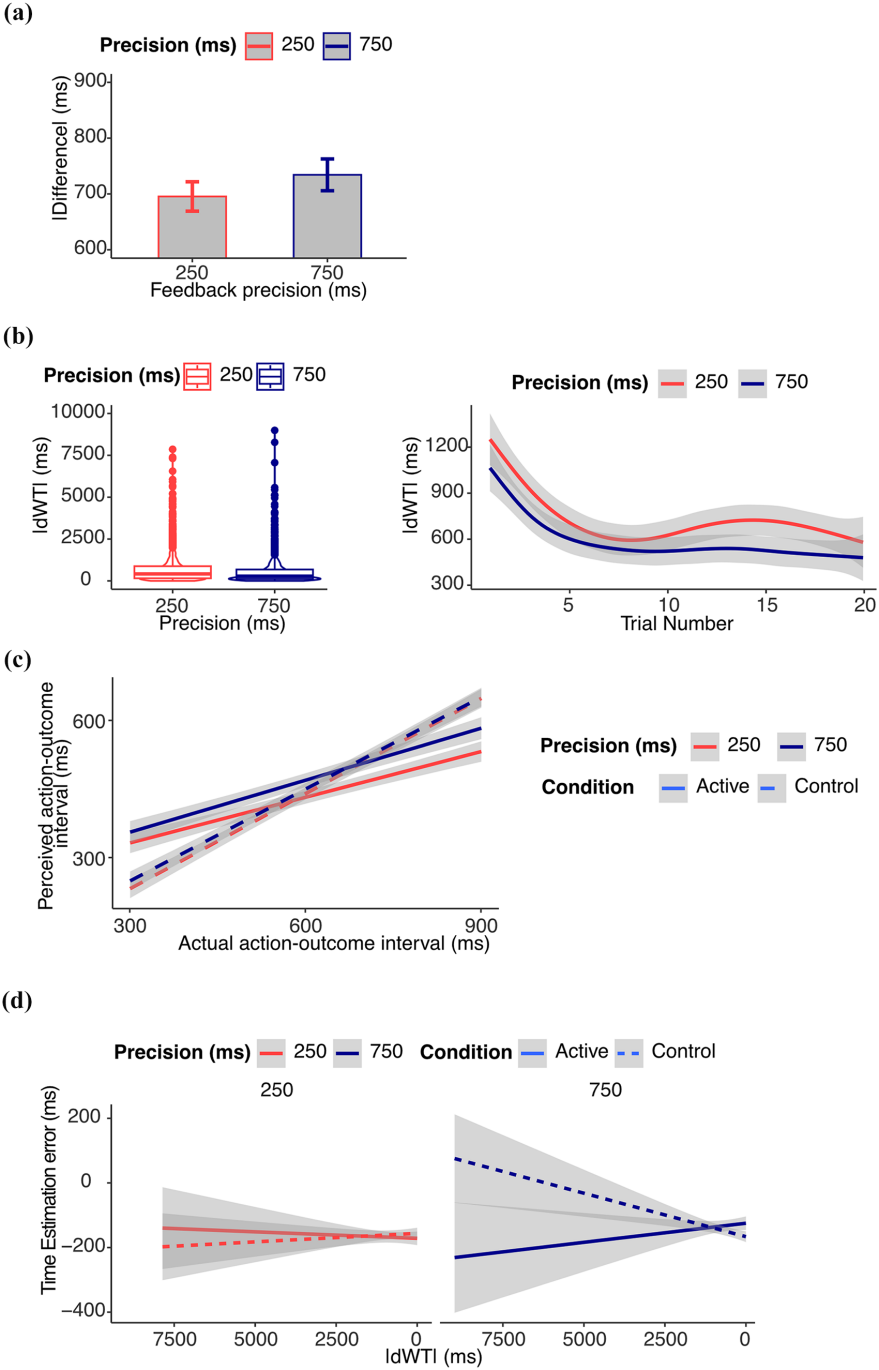
*Results from Experiment 2*. (a) *Performance*: absolute difference (ms) between waiting time and target waiting time. Figure shows that the untransformed data and error bars represent within-subject standard errors, computed using the standard error of paired differences. Statistical results refer to log-transformed data. (b) *Learning*: Learning progress measured as the absolute change in waiting time (|dWT|) from one trial to the next. Higher values indicate larger trial-to-trial adjustments (exploration), while lower values suggest more stable estimates (exploitation). Figure shows the untransformed data, statistical results refer to log-transformed data. (c) *IB*: estimated action–outcome interval. The perceived action–outcome intervals were shorter in the active than in the control condition. (d) *Relation between IB and learning*: time estimation error (difference between estimated and actual delay). In the active condition, the IB effect decreased with learning measured as |dWT|. The effect was statistically significant only in the 750 ms feedback precision condition.

Results from supplementary analyses on performance are reported in the Supplemental Materials and in Figure 3a.

#### Learning

Results from supplementary analyses on |dWT| are reported in the supplementary materials and in Figure 3b.

#### Intentional binding

A significant three-way interaction between condition, |dWT|, and feedback precision was again found (β = .071, *SE* = 0.026, 95% CI [0.021, 0.122], χ^2^(1) = 12.26, *p* < .001). Post hoc comparisons showed that the reduction of the IB effect with learning was greater in the active than control condition only in the 750 ms feedback precision condition (*p* = .0022).

The slope was significantly less than zero in the active 750 ms feedback precision condition (β = –.025 (*SE* = 0.010), 95% CI [–0.044, –0.007], χ^2^(1) = 6.96, *p* = .008), and significantly greater than zero in the control 750 ms feedback precision condition (β = .020 (*SE* = 0.008), [0.005, 0.036], χ^2^(1) = 6.60, *p* = .010). The results are reported in Figure 3c and d. All the results from the model exploration are reported in the Supplemental Materials.

## Experiment 3

In experiments 1 and 2, the IB measure implied judging the interval between action and tone, but the tone had no relevance to the primary task of trying to act at the right time. Indeed, the outcome tone was incidental to the learning task. IB reduction with learning might be different when IB was measured with respect to the actual feedback used to update performance. Therefore, in Experiment 3, we investigated whether the relevance of the action outcome influences the relation between learning and IB. To do this we again asked people to judge the interval between action and tone, but we paired the tone with the visual feedback signal that providing information about waiting time, and guided action selection. We hypothesized here that the IB reduction with learning would be smaller when the IB relates to action outcomes that are relevant for performance monitoring and improvement.

### Materials and methods

#### Participants

An independent sample of 30 participants (9 female, mean age ± *SD*: 25.7 ± 3.6 years) took part in this experiment. Three participants were excluded from the analyses in compliance with the participants’ screening procedure (see Supplemental Materials).

#### Task

**Figure 4.**
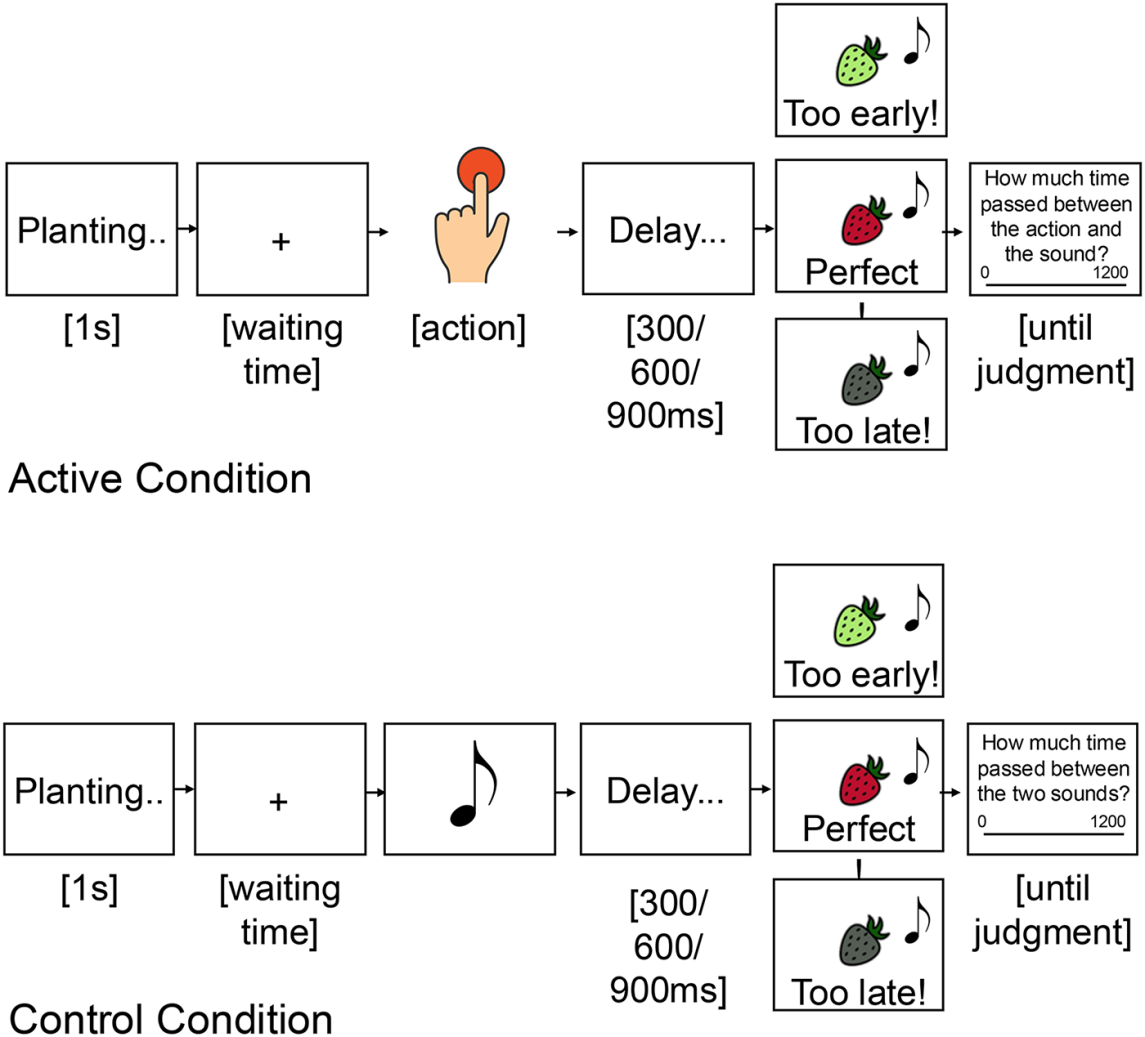
*Temporal reinforcement learning task (Experiment 3)*. A seed was planted in the ground at the start of each trial, and participants had to wait an unknown time before pressing a button to harvest it. They also had to estimate the delay between the time of their button press (or time of a first sound in the control condition (b) ) and the time of the following sound. The sound was presented together with feedback on participants’ waiting time.

The task used in Experiment 3 replicated those of experiments 1 and 2. The crucial difference regarded the timing of action feedback, that is, feedback about the time participants waited before pressing the spacebar to harvest the fruit/vegetable. While in experiments 1 and 2, participants saw action feedback *after* the tone and after their temporal judgment, in Experiment 3, feedback was provided at the same time as the tone, and thus *before* the temporal judgment. For a graphical representation of the task, please see Figure 4.

#### Data analysis

The analyses for Experiment 3 exactly replicated those of experiments 1 and 2. Trials in which reaction times exceeded ± 3 *SD* (0.6%) were discarded from the analyses.

To assess the potential influence of feedback valence on IB, we conducted exploratory mixed-effects models including feedback value as a predictor. Condition (Active/Control) was also entered as a fixed effect, and Participant as a random intercept. This analysis is reported in the Supplemental Materials.

Data and scripts are available at the following link: https://osf.io/w7hs9/?view_only=df9ee62f3e0942c39c0ea8c57388ac19

### Results

#### Performance

**Figure 5.**
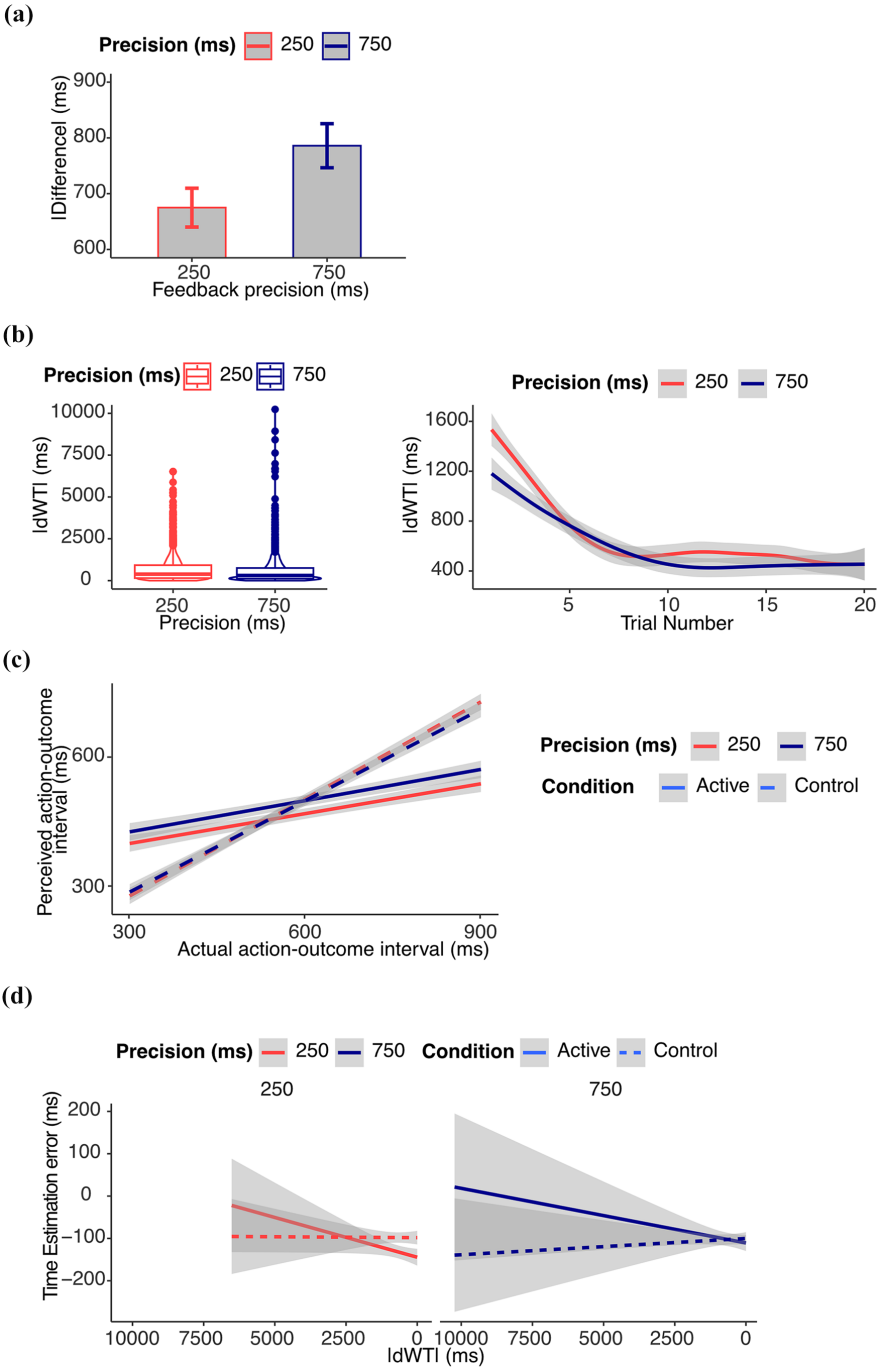
*Results from Experiment 3*. (a) *Performance*: absolute difference (ms) between waiting time and target waiting time. Figure shows the untransformed data and error bars represent within-subject standard errors, computed using the standard error of paired differences. Statistical results refer to log-transformed data. (b) *Learning*: Learning progress measured as the absolute change in waiting time (|dWT|) from one trial to the next. Higher values indicate larger trial-to-trial adjustments (exploration), while lower values suggest more stable estimates (exploitation). Figure shows the untransformed data, statistical results refer to log-transformed data. (c) *IB*: estimated action–outcome interval. The perceived action–outcome intervals were shorter in the active than in the control condition. (d) *Relation between IB and learning*: time estimation error (difference between estimated and actual delay). The IB effect did not decrease with learning measured as |dWT| in any experimental condition.

Results from supplementary analyses on performance are reported in the supplementary materials and in Figure 5a.

#### Learning

Results from supplementary analyses on |dWT| are reported in the Supplemental Materials and in Figure 5b.

#### Intentional binding

We found a nonsignificant interaction between |dWT|, condition, and feedback precision (β = –.03 (*SE* = 0.02), 95% CI [–0.08, 0.02], χ^2^(1) = 0.004, *p* = .95. The results are reported in Figure 5c and d. All the results from the model exploration are reported in the Supplemental Materials.

## Experiment 4

In a fourth experiment, we aimed to replicate the result of experiments 1, 2, and 3 in a within-subject design.

We directly compare the effect of learning on IB for incidental and instrumental events. In line with the results from experiments 1 and 2, we expected the IB for incidental events to reduce with learning, particularly in the 750 ms feedback precision condition. Conversely, the results of experiment 3 would imply no relation between learning and IB in instrumental conditions where action outcomes are paired with action feedback.

### Materials and methods

#### Participants

An independent sample of 30 participants (10 female, mean age ± *SD*: 26.1 ± 4.8 years) took part in this experiment. Five participants were excluded from the analyses in compliance with the participants’ screening procedure (see Supplemental Materials).

#### Task

The experimental paradigm consisted of a combination of experiments 1 and 2 and experiment 3, but we added the factor “Outcome Relevance.”

In some blocks, participants judged the delay between the time of their action (i.e., keypress) and the time of a tone following the action after a random delay (as in experiments 1 and 2). Feedback about the time participants waited before pressing the spacebar to harvest the fruit/vegetable was provided after the temporal judgment. This condition, where the tone was not relevant for guiding performance, was labelled “Incidental Outcome.”

In other blocks, participants judged the delay between the time of their action and the time of a compound action outcome following the action after a random delay. This compound action outcome consisted of a tone and the action feedback presented simultaneously (as in Experiment 3). Because the tone was relevant for guiding action selection, this condition was labelled “Instrumental Outcome.”

**Figure 6.**
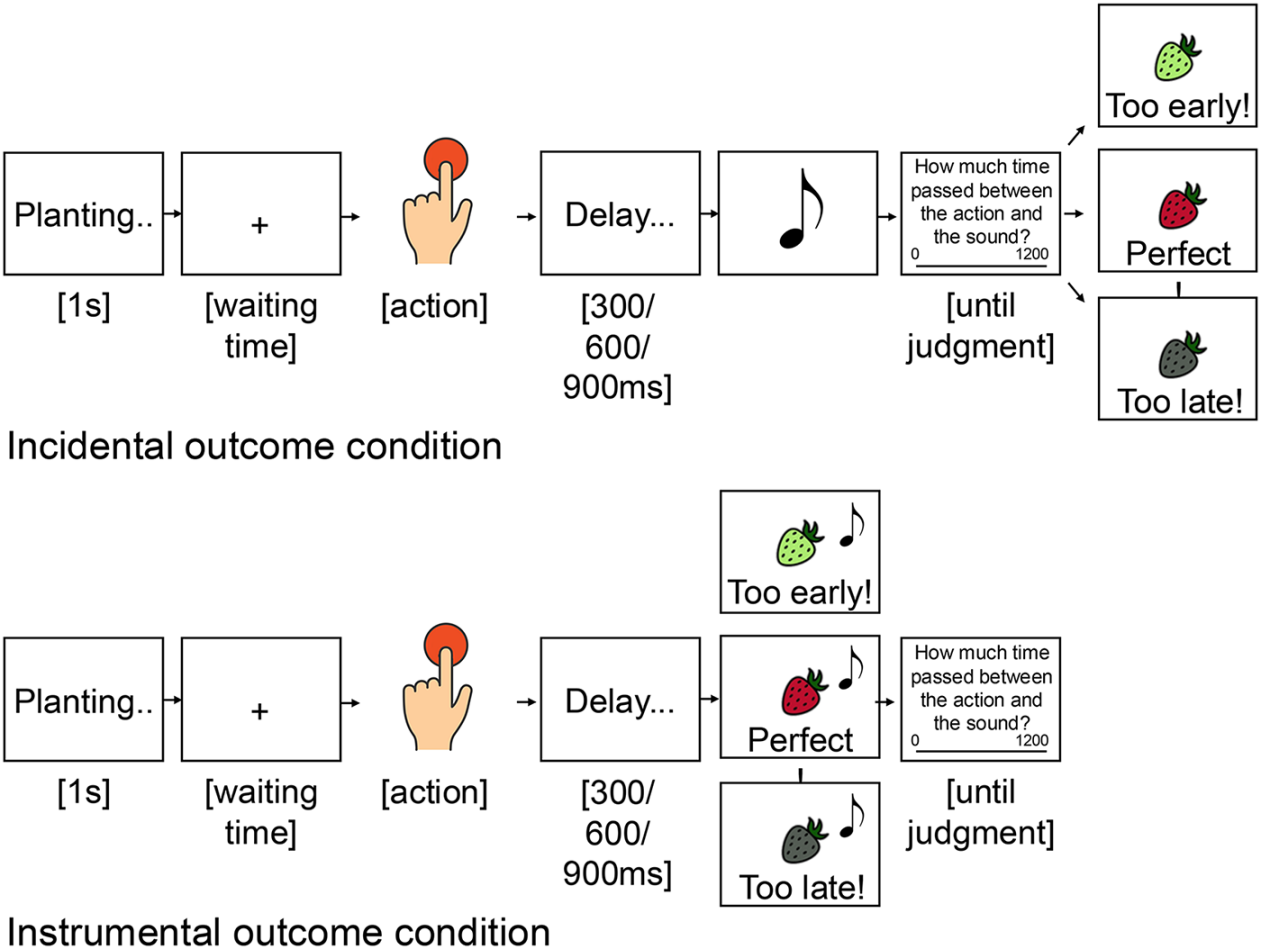
*Temporal reinforcement learning task (Experiment 4)*. In the incidental outcome condition (a), participants estimated the temporal delay between their button press and the following sound. Feedback on the waiting time was provided after the temporal judgment. In the instrumental outcome condition (b), participants estimated the temporal delay between their button press and the sound. However, in this condition, feedback on waiting time was presented simultaneously with the sound, making the outcome relevant for performance monitoring.

No control condition was included in this design, so all trials involved the participant’s active movement. For a graphical representation of the task, please see Figure 6.

#### Data analysis

The analyses for experiment 4 replicated those of experiments 1 and 2.

For the IB analysis, outcome relevance (incidental/instrumental outcome) was entered in the mixed model as a fixed effect instead of condition (active/control).

Trials in which reaction times exceeded ± 3 *SD* (0.7%) were discarded from the analyses.

To assess the potential influence of feedback valence on IB, we conducted exploratory mixed-effects models including feedback value as a predictor. Outcome relevance (instrumental/incidental) was also entered as a fixed effect, and participant as a random intercept. This analysis is reported in the Supplemental Materials.

Data and scripts are available at the following link: https://osf.io/w7hs9/?view_only=df9ee62f3e0942c39c0ea8c57388ac19

### Results

#### Performance

**Figure 7.**
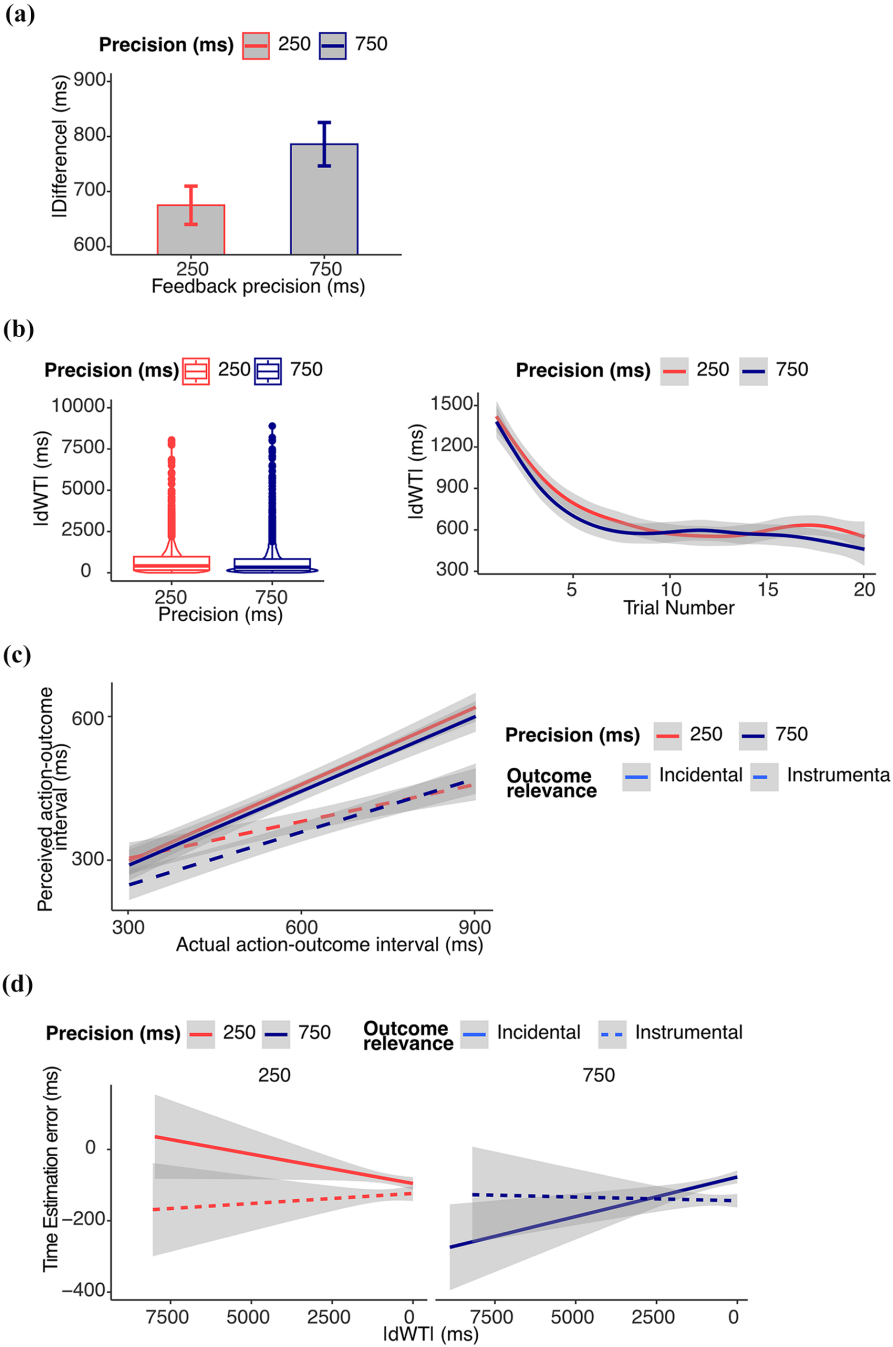
*Results from Experiment 4*. (a) *Performance*: absolute difference (ms) between waiting time and target waiting time. Figure shows the untransformed data and error bars represent within-subject standard errors, computed using the standard error of paired differences. Statistical results refer to log-transformed data. (b) *Learning*: Learning progress measured as the absolute change in waiting time (|dWT|) from one trial to the next. Higher values indicate larger trial-to-trial adjustments (exploration), while lower values suggest more stable estimates (exploitation). Figure shows the untransformed data, statistical results refer to log-transformed data. (c) *IB*: estimated action–outcome interval. The perceived action–outcome intervals were shorter in the incidental than in the instrumental outcome condition. (d) *Relation between IB and learning*: time estimation error (difference between estimated and actual delay). When the outcome was incidental, the IB effect decreased with learning, as measured by |dWT|. This effect was statistically significant only in the 750 ms feedback precision condition. When the outcome was instrumental, the IB effect did not decrease with learning, as measured by |dWT|, in any experimental condition.

Results from supplementary analyses on performance are reported in the Supplemental Materials and in Figure 7a.

#### Learning

Results from supplementary analyses on |dWT| are reported in the Supplemental Materials and in Figure 7b.

#### Intentional binding

We found a significant three-way interaction between outcome relevance, |dWT|, and feedback precision was significant (β = .062 (*SE* = 0.029), 95% CI [0.005, 0.119], χ^2^(1) = 4.96, *p* = .026). The interaction was explored by means of two separate analyses for each condition.

The analysis of the incidental outcome condition revealed a significant two-way interaction between |dWT| and feedback precision (β = –.0405, *SE* = 0.0102, 95% CI [–0.0605, –0.0205], χ^2^(1) = 15.76, *p* < .0001). When feedback precision was low (750 ms), the effect of |dWT| on the IB was negative and significant (β = –.0257, *SE* = 0.0072, [–0.0398, –0.0117], χ^2^(1) = 12.84, *p* = .0003). In other words: IB was reduced as participants learn the correct time to act. We note in passing that IB increased during learning in the 250 ms feedback precision condition (β = .0171, *SE* = 0.0080, [0.0013, 0.0328], χ^2^(1) = 4.50, *p* = 0.034). This result was not predicted. However, our experimental design focussed on differences between conditions, not on the amount of learning-related change in SoA in any single condition. Further, since trials with large updates are typically few in number, and occur very early in learning, this result could be driven by a small number of observations and should therefore be treated with caution. The remaining effects were not significant (all *p* values > .16).

The analysis of the instrumental outcome condition revealed no significant effects (all *p* values > .14).

The results are reported in Figure 7c and d.

## Discussion

In a temporal reinforcement learning paradigm, we asked participants to learn the best time to act. Actions were followed by an auditory outcome (i.e., a tone), and participants judged the time delay between their action (i.e., a keypress) and the generated outcome.

In experiments 1 and 2, after their temporal judgment, participants received signed error feedback on whether they had acted at the correct time, or too early or too late. We showed that the temporal linkage between the action and the subsequent self-generated sound (i.e., the IB effect) decreased as participants learned through experience how long to wait before acting. The IB was greater when participants explored and tried to select the best course of action to obtain the desired outcome, and updated their selection on successive trials. IB reduced when actions were updated to be more closely aligned to an internal model of when to act.

In both experiments 1 and 2, this learning effect on IB was absent in a control condition without voluntary action. We did not include a standard passive movement condition often used as a baseline in IB studies (the studies were done under social distancing rules during the COVID-19 pandemic). Instead, we used a control condition in which participants judged the interval between two externally generated tones (Haggard et al., 2003). While this condition differs from the learning task in terms of both motor involvement and sensory modality, it nonetheless served as a necessary comparison to rule out generic changes in time estimation over the course of the block. The absence of any adaptation in the two-tone control condition supports the interpretation that the effect is specific to selection and execution of a voluntary movement, rather than a general adaptation in time estimation over the course of the experiment. However, the structural differences between the tasks—particularly in the bimodal nature of the temporal judgment in the agency condition—should be acknowledged when interpreting this control comparison.

Crucially, we showed that feedback precision influenced the relation between learning and the IB. The precision of the feedback was manipulated by drawing from a probability distribution centred on the block’s target time, but with a standard deviation of either 250 or 750 ms. A 750 ms feedback precision implied greater tolerance toward temporally imprecise button presses. Conversely, obtaining and maintaining positive feedback was more difficult in the 250 ms condition: any response time different than the optimal waiting time was likely to yield negative feedback. However, feedback in the 250 ms condition was more precise, and thus more informative for adapting future behavior. This experimental manipulation had three distinct consequences on the behavior.

First, the overall performance was better in the 250 ms than 750 ms feedback precision condition. This is because feedback in the 250 ms condition was more precise, and thus more informative for adapting future behavior. Consequently, the difference between participants’ waiting time and the optimal waiting time was greater in the 750 ms than 250 ms feedback precision condition. However, this latter result should be cautiously considered since it was inconsistent across experiments. Indeed, we found an overall better performance in the 250 ms feedback precision condition in experiments 2, 3, and 4, and steeper difference reduction over time in the 250 ms feedback precision condition in experiment 1. However, experiment 2 showed a steeper difference reduction in the 750 ms feedback precision. Yet, this seems to be due to a greater difference in the 750 ms condition at the beginning of the experiment rather than to a greater improvement over time.

Second, we observed an overall smaller trial by trial update (|dWT|) in the 750 ms than 250 ms feedback precision condition across all the experiments. More precise feedback led to a better performance, but less precise feedback led to more “correct” trials, and a tendency to exploit rewarded behavior by acting at the same waiting time on each trial. Therefore, in the 750 ms feedback precision condition, more stable positive feedback determined smaller |dWT| compared to the 250 ms feedback precision condition.

Crucially, we found a significant reduction of the IB effect with learning only in the low precision feedback condition (750 ms). This seems to suggest that the IB reduction with learning was driven by participants’ transition from exploration to exploitation rather than performance objective improvement. With learning, participants’ task switch from the need to acquire a model of when to act to using such a model to fluently produce the desired outcome. We suggest that in the 750 ms feedback precision condition, consistently positive feedback accompanying participants’ performance facilitated the transition from exploration to exploitation behavior, as evident from smaller trial by trial updates (|dWT|) when feedback was less precise. In other words, stable positive feedback in the low precision condition might have given participants a feeling of succeeding in the task, even though this was not necessarily accompanied by more accurate action selection. Conversely, in the 250 ms feedback precision condition, the greater precision of feedback (and the consequent large number of errors) counteracts the tendency to shift from exploration to exploitation, so that action updating remains strong, cognitive effort and control continue to be required, and IB does not reduce with learning to the same extent. This is also in line with the results from Wen and Haggard (2020). They showed that the sense of control is better preserved in contexts where the environment behaves in a stable and predictable manner, even if performance is not optimal. In their task, participants experienced a higher sense of control when the sensorimotor disturbance was systematic and could be learned over time, compared to when the disturbance was random. Similarly, in our study, the low precision feedback condition may have provided a stable context that supported the consolidation of internal action–outcome models and promoted a shift toward automatic, fluent performance—despite the feedback being less informative in objective terms.

We showed that as action selection becomes routine, automatized, and unchallenging (as particularly in our low-precision feedback condition), the IB effect reduces. This hypothesis might also explain why IB did not reduce when the outcome was epistemically relevant. In experiments 3 and 4, we investigated the case where the outcome event used for measuring IB was associated with feedback that could guide performance. IB did not then decrease with learning. We argue that the epistemic value of the outcome event leads to a requirement to continually monitor action–outcome linkage. When the outcome remains for whatever reason important, the experienced linkage between action and outcome remains strong (experiments 3 and 4). Conversely, when actions are overlearned, and the action–outcome linkage is irrelevant to the task (experiments 1 and 2) the experience of action and outcome as linked in time becomes less salient.

This study showed that IB reduced with learning. Although the association between IB and sense of agency has at times been questioned (Kirsch et al., 2019; Kong et al., 2024; Schwarz et al., 2019; Siebertz & Jansen, 2022), we suggest that IB has some value as a proxy measure for agency-related processes (Galang et al., 2021; Imaizumi & Tanno, 2019; Pyasik et al., 2018), and therefore interpret our findings in line with this idea. If IB is used as a proxy measure for agency-related processes, then our results suggest that the sense of agency decreases with learning. Importantly, this should not be interpreted as a total abolition of agency, but rather as a relative reduction over time. In the early stages of learning, participants may engage more attentively and effortfully with the task, closely monitoring their actions and outcomes to develop a model of when to act. This heightened cognitive engagement may temporarily elevate the experience of agency. As learning progresses and actions become more automatic, the need for such close monitoring diminishes. In this context, the observed IB reduction may either reflect a return to a more stable or baseline level of agency, or a genuine attenuation of the experience over time. Since there is no established “ground truth” or objective baseline for the sense of agency, our interpretation is based on tracking dynamic changes during a defined learning process, rather than assuming a fixed reference point. This view is compatible with the idea that agency fluctuates according to task demands, cognitive control, and outcome relevance, rather than being a static property of action execution.

Our results are in line with the studies showing that IB is associated with difficulty, feedback-guided adaptation, and the process of updating action values (Di Costa et al., 2017; Majchrowicz et al., 2020). However, these findings differ from those of Pansardi et al. (2020), who reported that lifelong musical training enhances IB. While their results suggest that long-term sensorimotor training enhances action–outcome integration, our findings show that IB can decrease over the course of a short-term learning session. This apparent discrepancy may reflect differences in the type and timescale of learning involved. In Pansardi et al. (2020), participants had developed highly refined action-effect models over years of practice, which may foster more stable predictions of action outcomes. By contrast, our study involved participants navigating uncertainty and receiving probabilistic feedback over the course of a single session. As learning progressed and actions became routine, IB declined—perhaps reflecting a reduced need to monitor action–outcome associations once the task became predictable and automatic. Thus, the difference in findings may stem from a shift in the function of agency-related processes—from effortful monitoring during early learning to implicit control in skilled performance—highlighting distinct underlying mechanisms across short- and long-term learning contexts (Haith & Krakauer, 2013).

Our finding that learning-related changes in action performance were associated with reduced IB contrasts with prior reports that action selection fluency can enhance explicit sense of agency (Chambon & Haggard, 2012; Sidarus et al., 2013). One possible explanation is that the cognitive processes underlying fluency differed between studies: the subliminal priming used by Chambon et al. may boost agency experiences by enhancing immediate action selection, while learning and adaptation may reduce attentional engagement or prediction error monitoring, leading to reduced temporal binding. Alternatively, this discrepancy may reflect the broader theoretical debate about the relationship between IB and explicit agency judgments (Galang et al., 2021; Imaizumi & Tanno, 2019; Pyasik et al., 2018; Schwarz et al., 2019; Siebertz & Jansen, 2022).

This study has several limitations that should be acknowledged. First, we relied on IB as a proxy measure for agency-related processes. While IB has been shown to be sensitive to factors that might plausibly contribute to sense of agency, such as intentionality and free choice (Beck et al., 2017; Borhani et al., 2017; Caspar et al., 2016; Engbert & Wohlschläger, 2007), recent work has questioned its value as a measure of agency (Kirsch et al., 2019; Kong et al., 2024; Suzuki et al., 2019). In particular, IB effects have been observed even in the absence of voluntary action, suggesting that it may reflect broader mechanisms such as temporal prediction or multisensory integration. Although no single measure can fully capture the complex, subjective experience of agency, and explicit reports are themselves prone to biases, future work could benefit from combining IB with additional explicit measures to provide a more comprehensive assessment of agency-related processes. It is useful to consider the specific contrasts involved in each IB experiment, and to consider whether the factor operationalized in that contrast is a plausible component of sense of agency, or not.

Second, the task structure inherently involved dual cognitive demands: participants had to learn when to act to maximize positive feedback (temporal reinforcement learning), while also performing time estimations for the IB measure. Although this dual-task structure was a necessary feature of the design—enabling trial-by-trial associations between learning and IB—it may have introduced cognitive resource competition. Importantly, however, the reduction in IB over time was only observed when feedback was delayed (experiments 1 and 2), not when it was presented concurrently with the outcome tone (experiments 3 and 4). This suggests that the findings cannot be fully attributed to general cognitive load or overlapping timing demands, and instead point to a specific interaction between learning and outcome relevance.

In conclusion, we showed that the IB decreased as participants learned through experience how long to wait before acting. However, this reduction seems to occur only in specific conditions. First, it does not occur when learning is not accompanied by stable positive feedback signalling the skill acquisition. Second, it does not occur when the outcome of action continues to demand attention because of its relevance to the task. Thus, when an action–outcome link is relevant and important, IB tends to remain high, and not reduce with learning.

## Supplemental Material

sj-docx-1-qjp-10.1177_17470218251349521 – Supplemental material for Intentional binding decreases during learning: Implications for sense of agency Supplemental material, sj-docx-1-qjp-10.1177_17470218251349521 for Intentional binding decreases during learning: Implications for sense of agency by Silvia Seghezzi, Elisabeth Parés-Pujolràs and Patrick Haggard in Quarterly Journal of Experimental Psychology
