# Supplementary material for: Intentional binding decreases during learning: Implications for sense of agency
Source: Q J Exp Psychol (Hove). 2025 Jun 2;79(3):612–29. doi: 10.1177/17470218251349521 (PMC12901642; doi:10.1177/17470218251349521)
Supplement: sj-docx-1-qjp-10.1177_17470218251349521 – Supplemental material for Intentional binding decreases during learning: Implications for sense of agency [file sj-docx-1-qjp-10.1177_17470218251349521.docx]

**Supplementary Materials**

# **Experiment 1**

## Participants screening

Due to the difficulties of assuring participants’ compliance through online platforms for data acquisition, we introduced two compliance assessments, to check participants’ learning and participants’ ability in time estimation, respectively.

To check for learning, we computed (for each participant) the rank correlation between the trial number and |dWT|. A negative rho coefficient indicates a progressive reduction of adjustments in the motor plan throughout the task. Based on this index, we sought to exclude from analyses participants showing the inverse pattern, i.e., a positive association between trial number and |dWT|. As far as experiment 1 is concerned, none of the participants was excluded from analyses based on this criterion. See **Figure S1A**.

Similarly, to check for participants’ overall accuracy in the time estimation judgements, for each participant, we computed the rank correlation between the experimentally manipulated delay and the reported interval estimation. A positive rho coefficient indicates that - as the modulated action-outcome interval increases - the participant’s judgement of the elapsed time increases as well. Based on this index, we sought to exclude from analyses participants showing a negative association between action-outcome delay and the estimated time interval (overall longer estimations for shorter action-outcome delays and vice versa). As far as experiment 1 is concerned, none of the participants was excluded from analyses based on this criterion. See **Figure S1B**.

**Figure S1: A.** Single-subject visualisation of the rank correlation between trial number and the absolute trial-to-trial changes in waiting times (|dWT| (ms)). Green lines represent linear regression fits computed using least squares (ggplot2’s geom_smooth(method = "lm")). A negative slope indicates a progressive reduction in adjustments over trials, consistent with learning. **B**. Single-subject visualisation of the rank correlation between the experimentally manipulated action-outcome interval (ms) and the estimated action-outcome interval (ms).

## Supplementary analyses: Performance

*Methods*

We computed the unsigned difference between participants’ waiting time on each trial and the target waiting time for that block (|Difference|). The data were log transformed to obtain a better approximation to normal distribution. Performance was analysed in a mixed-effects model analysis with trial number and feedback precision (250/750 ms) as fixed effects. We predicted that difference values would decrease across each block due to learning, and that more precise feedback would allow more learning.

*Results*

A linear mixed-effects model was fitted to predict log-transformed |Difference| values based on trial number, feedback precision, and their interaction. The model explained a small to moderate proportion of variance (marginal R² = .10; conditional R² = .17).

There was a significant main effect of trial number, (β = -0.078 (SE = 0.005), 95% CI [-0.088, -0.069], χ²(1) = 398.40, p < .001), indicating that absolute difference values decreased over trials, consistent with learning.

The main effect of feedback precision was not significant, (β = -0.081 (SE = 0.083), 95% CI [-0.245, 0.082], χ²(1) = 2.19, p = .139). Absolute difference values were comparable between the 250-ms feedback precision condition (mean = 768.58 ms, SD = 1494.87) and the 750-ms feedback precision condition (mean = 805.75 ms, SD = 1599.42). The results are reported in **Figure 2a** in the main manuscript.

The two-way interaction between trial number and feedback precision was significant, (β = 0.015 (SE = 0.007), 95% CI [0.001, 0.028], χ²(1) = 4.14, p = .042). This interaction revealed a steeper improvement (greater decrease in |Difference| across trials) in the 250-ms feedback precision condition compared to the 750-ms condition.

## Supplementary analyses: Learning

*Methods*

To check the validity of the experimental manipulation, |dWT| was entered as the dependent variable in a mixed-effects model analysis with trial number as a fixed effect. To explore any differences in |dWT| due to the feedback precision condition, feedback precision (250/750ms) was also entered in the model as a fixed effect. Subjects (ID) were modelled as random intercepts. Logarithmic transformation was adopted to obtain a better approximation to a Normal distribution than that of raw |dWT|.

We expected strong updating initially, and weaker updating later on in each block. We also expected more updating for higher precision feedback (250 vs 750ms). This is because a wider tolerance window results in more positive feedback, and positive feedback does not lead to adjustments in performance.

#### Results

A linear mixed-effects model was fitted to predict log-transformed |dWT| values based on trial number, feedback precision, and their interaction. The model explained a small proportion of variance (marginal R² = .07; conditional R² = .13).

There was a significant main effect of trial number, (β = -0.063 (SE = 0.006), 95% CI [-0.075, -0.052], χ²(1) = 222.80, p < .001), indicating that |dWT| values decreased across trials, consistent with standard models of learning.

A significant main effect of feedback precision was also found, (β = -0.276 (SE = 0.098), 95% CI [-0.469, -0.083], χ²(1) = 24.95, p < .001), with greater |dWT| values observed in the 250-ms feedback condition (mean = 847.43 ms, SD = 220.10) compared to the 750-ms feedback condition (mean = 727.36 ms, SD = 234.78).

The interaction between trial number and feedback precision was not significant, (β = 0.004 (SE = 0.008), 95% CI [-0.012, 0.020], χ²(1) = 0.21, p = .648).

The results are reported in **Figure 2b** in the main manuscript.

## Supplementary results: intentional binding

A linear mixed-effects model was fitted to predict intentional binding (TC) based on action condition (active vs. control), feedback precision, performance error (|dWT|), delay, and their interactions. The model explained a moderate proportion of variance (marginal *R²* = .19; conditional *R²* = .45).

There was a significant main effect of condition, (β = -109.7 (SE = 18.5), 95% CI [-145.92, -73.50], χ²(1) = 53.29, *p* < .001), indicating greater binding in the active condition (mean = -149.1 ms, SD = 181.2) compared to the control condition (mean = -108.5 ms, SD = 157.2).

A significant main effect of |dWT| was found, (β = 0.006 (SE = 0.011), 95% CI [-0.014, 0.027], χ²(1) = 5.03, *p* = .025), showing that larger deviations were associated with stronger binding.

Feedback precision also had a significant main effect, (β = 42.54 (SE = 17.57), 95% CI [8.10, 76.98], χ²(1) = 9.56, *p* = .002), with stronger binding in the 250-ms feedback precision condition (mean = -137.6 ms, SD = 143.2) than in the 750-ms feedback precision condition (mean = -120.4 ms, SD = 163.2).

Delay had a significant main effect, (χ²(2) = 1488.27, p < .001). Post hoc comparisons revealed that binding was greater at 900-ms delay (mean = -261.7 ms, SD = 182.5) than at both 600-ms (mean = -125.4 ms, SD = 163.4) and 300-ms delays (mean = -1.22 ms, SD = 153.1), all p < .001, and greater at 600-ms than at 300-ms delay, p < .001.

The interaction between condition and |dWT| was significant, (β = -0.006 (SE = 0.015), 95% CI [-0.035, 0.024], χ²(1) = 9.92, *p* = .002).

The interaction between condition and delay was also significant, (χ²(2) = 530.46, *p* < .001).

Finally, a significant three-way interaction between condition, |dWT|, and feedback precision was found, (β = 0.017 (SE = 0.020), 95% CI [-0.023, 0.057], χ²(1) = 4.05, *p* = .044). Post hoc analyses showed that the slope of the |dWT| effect on binding was steeper in the active condition compared to the control condition under the 750-ms feedback precision setting (*p* = .002), and steeper in the active 750-ms condition compared to both the active 250-ms (*p* = .04) and control 250-ms conditions (*p* = .003). The remaining comparisons were not significant (*p* > .81).

The remaining comparisons were not significant (all p-values > 0.81). The results are reported in **Table S1** and in **Figure 2c and d** in the main manuscript.

**Table S1**: Post-hoc analyses results from the significant three-way interaction between condition, |dWT| and feedback precision.

| Contrast | Estimate | SE | Df | t.ratio | P-value |
| --- | --- | --- | --- | --- | --- |
| *active 250 – control 250* | -0.006 | 0.008 | 6117 | -0.81 | 0.85 |
| ***active 250 – active 750*** | **0.02** | **0.008** | **6118** | **2.68** | **0.037*** |
| *active 250 – control 750* | -0.007 | 0.008 | 6118 | -0.874 | 0.81 |
| *control 250 – active 750* | 0.028 | 0.008 | 6118 | 3.49 | 0.003 |
| *control 250 – control 750* | -0.0006 | 0.008 | 6118 | -0.07 | 0.99 |
| ***active 750 – control 750*** | **-0.028** | **0.008** | **6117** | **-3.55** | **0.002*** |

To further explore this effect, we performed separate analyses for 250ms feedback precision and 750ms feedback precision conditions.

The model on 250ms feedback precision condition explained a small proportion of variance (marginal *R²* = .006; conditional *R²* = .25).

There was a significant main effect of condition, (β = 37.33 (SE = 11.74), 95% CI [14.32, 60.34], χ²(1) = 24.98, *p* < .001, indicating greater binding in the active condition (mean = -160.0 ms, SD = 168.5) compared to the control condition (mean = -114.9 ms, SD = 154.9).

Neither the main effect of |dWT|, (β = -0.006 (SE = 0.006), 95% CI [-0.019, 0.006], χ²(1) = 0.21, *p* = .643), nor the interaction between performance error and condition, (β = 0.008 (SE = 0.009), 95% CI [-0.009, 0.026]), χ²(1) = 0.88, *p* = .348, were significant.

The model on 750ms feedback precision condition explained a small proportion of variance (marginal *R²* = .008; conditional *R²* = .30).

There was a significant main effect of condition, (β = 14.02 (SE = 11.19), 95% CI [-7.92, 35.96], χ²(1) = 15.83, *p* < .001), indicating greater binding in the active condition (mean = -138.6 ms, SD = 203.8) compared to the control condition (mean = -101.9 ms, SD = 161.8).

A significant main effect of |dWT| was observed, (β = -0.027 (SE = 0.007), 95% CI [-0.040, -0.014], χ²(1) = 6.42, *p* = .011), indicating that greater performance deviations were associated with stronger binding.

Importantly, a significant two-way interaction between |dWT| and condition was found, (β = 0.030 (SE = 0.009), 95% CI [0.012, 0.048], χ²(1) = 10.40, *p* = .001), indicating a steeper slope of the effect of |dWT| on intentional binding in the active than the control condition.

Finally, we explored the association between performance error (|dWT|) and intentional binding separately for each condition.

In the active 750-ms feedback precision condition, the slope was significantly less than zero, (β = -0.021 (SE = 0.007), 95% CI [-0.035, -0.008], χ²(1) = 9.32, *p* = .002), indicating that greater trial by trial updates were associated with stronger binding. The model explained a small proportion of variance (marginal *R²* = .004; conditional *R²* = .375).

In contrast, the slope was not significant in the active 250-ms feedback precision condition, (β = -0.005 (SE = 0.007), 95% CI [-0.018, 0.009], χ²(1) = 0.43, *p* = .513).

Similarly, no significant association between performance error and binding was found in the control 250-ms feedback precision condition, (β = 0.0002 (SE = 0.0054), 95% CI [-0.010, 0.011], χ²(1) = 0.002, p = .967), or in the control 750-ms feedback precision condition, (β = -0.003 (SE = 0.006), 95% CI [-0.014, 0.008], χ²(1) = 0.27, p = .605).

# Experiment 2

## Participants screening

As in Experiment 1, we computed (for each participant) the rank correlation between trial number and |dWT|. A negative rho coefficient indicates a progressive reduction of adjustments in the motor plan throughout the task. Based on this index, we sought to exclude from analyses participants showing the inverse pattern. Two participants (ID= 5 & ID=18) were excluded from analyses based on this criterion. See **Figure S2A**.

Similarly, to check for participants’ overall accuracy in the time estimation judgements, for each participant, we computed the rank correlation between the experimentally manipulated delay and the reported interval estimation. Based on this index, we sought to exclude from analyses participants showing a negative association between action-outcome delay and the estimated time interval. One participant (ID=21) was excluded from analyses based on this criterion. See **Figure S2B**.

**Figure S2: A.** Single-subject visualisation of the rank correlation between trial number and the absolute trial-to-trial changes in waiting times (|dWT| (ms)). Green lines represent linear regression fits computed using least squares (ggplot2’s geom_smooth(method = "lm")). A negative slope indicates a progressive reduction in adjustments over trials, consistent with learning. **B**. Single-subject visualisation of the rank correlation between the experimentally manipulated action-outcome interval (ms) and the estimated action-outcome interval (ms).

## Supplementary analyses: Performance

#### Methods

#### The analyses for Experiment 2 exactly replicated those of Experiment 1.

#### Results

A linear mixed-effects model was fitted to predict log-transformed |Difference| values based on trial number, feedback precision, and their interaction. The model explained a small to moderate proportion of variance (marginal R² = .03; conditional R² = .31). A significant main effect of trial number was observed (β = –0.028, SE = 0.005, 95% CI [–0.038, –0.017], χ²(1) = 95.64, p < 0.0001), indicating greater |Difference| values at earlier trials. Feedback precision also showed a significant effect (β = 0.313, SE = 0.088, 95% CI [0.140, 0.487], χ²(1) = 7.58, p = 0.006), with worse performance in the 750 ms condition (mean = 771.36, SD = 606.53) than in the 250 ms condition (mean = 747.21, SD = 743.45). A significant interaction between trial number and feedback precision was found (β = –0.019, SE = 0.008, 95% CI [–0.034, –0.004], χ²(1) = 6.16, p = 0.013), indicating a steeper improvement in performance over trials in the 750 ms condition compared to the 250 ms condition. These results are illustrated in Figure **3a**.

## Supplementary analyses: Learning

#### Methods

#### The analyses for Experiment 2 exactly replicated those of Experiment 1.

#### Results

A linear mixed-effects model was fitted to predict log-transformed |dWT| values based on trial number, feedback precision, and their interaction. The model explained a small proportion of variance (marginal R² = .02; conditional R² = .15). A significant main effect of trial number was observed (β = –0.029, SE = 0.006, 95% CI [–0.041, –0.017], χ²(1) = 37.48, p < 0.0001), indicating greater |dWT| values at earlier trials. Feedback precision also had a significant effect (β = –0.303, SE = 0.104, 95% CI [–0.506, –0.099], χ²(1) = 25.61, p < 0.0001), with greater |dWT| in the 250 ms condition (mean = 745.30, SD = 420.68) than in the 750 ms condition (mean = 607.52, SD = 353.64). The interaction between trial number and feedback precision was not significant (β = 0.005, SE = 0.009, 95% CI [–0.012, 0.022], χ²(1) = 0.29, p = 0.59). Results are illustrated in **Figure 3b**.

## Supplementary results: intentional binding

A linear mixed-effects model was fitted to predict intentional binding (TC) based on performance error (|dWT|), action condition (active vs. control), feedback precision, delay, and all their interactions. The model explained a moderate proportion of variance (marginal R² = .20; conditional R² = .33).
A significant main effect of delay was observed (χ²(2) = 1367.80, *p* < .0001). Post-hoc comparisons showed that intentional binding was significantly greater for 900 ms (M = –304.43, SD = 174.59) than 600 ms (M = –139.08, SD = 121.76; estimate = 164, SE = 7.95, *z* = 20.58, *p* < .0001) and 300 ms (M = –9.52, SD = 89.88; estimate = 291, SE = 7.92, *z* = 36.73, *p* < .0001), and 600 ms was greater than 300 ms (estimate = 127, SE = 7.96, *z* = 16.01, *p* < .0001).
We also found a significant main effect of feedback precision (β = 43.10, SE = 19.60, 95% CI [4.68, 81.52], χ²(1) = 12.84, *p* = .0003), with greater intentional binding in the 250 ms condition (M = –142.07, SD = 112.84) than in the 750 ms condition (M = –163.49, SD = 107.62).
The main effects of |dWT| (β = 0.021, SE = 0.012, 95% CI [–0.003, 0.045], χ²(1) = 0.46, *p* = .50) and action condition (β = –99.10, SE = 20.08, 95% CI [–138.45, –59.75], χ²(1) = 0.58, *p* = .45) were not significant.

A significant two-way interaction between condition and feedback precision was observed (β = –52.10 (SE = 27.76), 95% CI [–106.52, 2.31], χ²(1) = 3.89, *p* = .049). Additionally, the interaction between |dWT| and delay was significant (χ²(2) = 19.34, *p* < .0001). The two-way interaction between condition and delay was significant (χ²(2) = 181.8, p<0.0001).

Crucially, a significant three-way interaction between |dWT|, condition, and feedback precision was found (β = 0.071, SE = 0.026, 95% CI [0.021, 0.122], χ²(1) = 12.26, p < .001). Post-hoc comparisons revealed a significantly steeper positive slope of the effect of |dWT| on intentional binding in the control compared to the active condition at 750 ms feedback precision (p = .0022), and a significantly shallower slope in the active condition at 750 ms compared to 250 ms feedback precision (p = .002). The remaining contrasts were not significant (all p > .23).

The results are reported in **Table S2** and in **Figure 3c and d** in the main manuscript.

**Table S2**: Post-hoc analyses results from the significant three-way interaction between condition, |dWT| and feedback precision.

| Contrast | Estimate | SE | Df | t | P-value |
| --- | --- | --- | --- | --- | --- |
| *active 250 – control 250* | 0.012 | 0.009 | 5299 | 1.26 | 0.58 |
| *active 250 – active 750* | 0.019 | 0.01 | 5300 | 1.85 | 0.25 |
| *active 250 – control 750* | -0.019 | 0.01 | 5300 | -1.88 | 0.23 |
| *control 250 – active 750* | 0.0068 | 0.01 | 5300 | 0.66 | 0.91 |
| *control 250 – control 750* | -0.032 | 0.01 | 5300 | -3.07 | 0.012 |
| ***active 750 – control 750*** | **-0.038** | **0.01** | **5299** | **-3.55** | **0.002*** |

To further explore this effect, we performed separate analyses for 250ms feedback precision and 750ms feedback precision conditions.

The model on 250ms feedback precision condition explained a negligible proportion of variance (marginal R² = .0005; conditional R² = .14). No significant effects were found: neither the main effect of |dWT| (β = 0.004 SE = 0.008, 95% CI [–0.011, 0.020], χ²(1) = 0.01, p = .92), the main effect of condition (β = 16.02 SE = 12.88, 95% CI [–9.21, 41.26], χ²(1) = 0.78, p = .38), nor their interaction (β = –0.010 SE = 0.011, 95% CI [–0.031, 0.012], χ²(1) = 0.79, p = .37) reached significance.

The model on 750ms feedback precision condition explained a small proportion of variance (marginal R² = .004; conditional R² = .15). A significant two-way interaction between |dWT| and condition was found (β = 0.038 SE = 0.012, 95% CI [0.014, 0.062], χ²(1) = 9.42, p = .002), indicating a steeper negative slope of the effect of |dWT| on intentional binding in the active compared to the control condition. Specifically, the effect of |dWT| was negative in the active condition (β = –0.021, SE = 0.009, 95% CI [–0.039, –0.004], p = .018), and the interaction term reversed the direction of the slope in the control condition.

Finally, we tested the slopes of the effect of |dWT| on intentional binding separately for each condition. The slope was significantly less than zero in the active 750 ms feedback precision condition (β = –0.025 (SE = 0.010), 95% CI [–0.044, –0.007], χ²(1) = 6.96, p = .008), and significantly greater than zero in the control 750 ms feedback precision condition (β = 0.020 (SE = 0.008), 95% CI [0.005, 0.036], χ²(1) = 6.60, p = .010). In contrast, the slope was not significantly different from zero in the active 250 ms condition (β = 0.004 (SE = 0.009), 95% CI [–0.013, 0.021], χ²(1) = 0.22, p = .64) or in the control 250 ms condition (β = –0.005 (SE = 0.007), 95% CI [–0.019, 0.009], χ²(1) = 0.56, p = .46).

Experiment 3

#### Participants screening

As in Experiments 1 and 2, we computed (for each participant) the rank correlation between trial number and |dWT|. A negative rho coefficient indicates a progressive reduction of adjustments in the motor plan throughout the task. Based on this index, we sought to exclude from analyses participants showing the inverse pattern. Three participants (ID= 7, ID=10 & ID=24) were excluded from analyses based on this criterion. See **Figure S3A**.

Similarly, to check for participants’ overall accuracy in the time estimation judgements, for each participant, we computed the rank correlation between the experimentally manipulated delay and the reported interval estimation. Based on this index, we sought to exclude from analyses participants showing a negative association between action-outcome delay and the estimated time interval. No participants were excluded from analyses based on this criterion. See **Figure S3B**.

**Figure S3: A.** Single-subject visualisation of the rank correlation between trial number and trial-to-trial changes in waiting times |dWT|. Green lines represent linear regression fits computed using least squares (ggplot2’s geom_smooth(method = "lm")). A negative slope indicates a progressive reduction in adjustments over trials, consistent with learning. **B.** Single-subject visualisation of the rank correlation between the experimentally manipulated action-outcome interval (ms) and the estimated action-outcome interval (ms).

## Supplementary analyses: Performance

#### Methods

#### The analyses for Experiment 3 exactly replicated those of Experiments 1 and 2.

#### Results

A linear mixed-effects model was fitted to predict log-transformed |Difference| values based on trial number, feedback precision, and their interaction. The model explained a moderate proportion of variance (marginal R² = .12; conditional R² = .17).

There was a significant main effect of trial number, β = -0.079 (SE = 0.005), 95% CI [-0.089, -0.069], χ²(1) = 463.42, p < .001, indicating that absolute deviation values decreased across trials, consistent with learning.

A significant main effect of feedback precision was also found, β = 0.131 (SE = 0.084), 95% CI [-0.034, 0.297], χ²(1) = 13.69, p < .001, showing greater |Difference| values in the 750-ms feedback precision condition (mean = 786.88 ms, SD = 1616.21) compared to the 250-ms feedback precision condition (mean = 676.18 ms, SD = 1431.32).

The two-way interaction between trial number and feedback precision was not significant, β = 0.003 (SE = 0.007), 95% CI [-0.011, 0.017], χ²(1) = 0.17, p = .678.

The results are reported in **Figure 5a** in the main manuscript.

## Supplementary analyses: Learning

#### Methods

#### The analyses for Experiment 3 exactly replicated those of Experiments 1 and 2.

#### Results

A linear mixed-effects model was fitted to predict log-transformed |dWT| values based on trial number, feedback precision, and their interaction. The model explained a small proportion of variance (marginal *R²* = .08; conditional *R²* = .16).

There was a significant main effect of trial number, β = -0.069 (SE = 0.005), 95% CI [-0.080, -0.058], χ²(1) = 278.27, *p* < .001, indicating greater |dWT| values for smaller trial numbers.

A significant main effect of feedback precision was also observed, β = -0.307 (SE = 0.093), 95% CI [-0.489, -0.125], χ²(1) = 22.16, *p* < .001, showing that |dWT| values were greater in the 250-ms feedback precision condition (mean = 678.17 ms, SD = 263.38) compared to the 750-ms feedback precision condition (mean = 603.36 ms, SD = 274.13).

The interaction between trial number and feedback precision was not significant, β = 0.009 (SE = 0.008), 95% CI [-0.006, 0.024], χ²(1) = 1.42, *p* = .234.

These results are reported in **Figure 5b** of the main manuscript.

## Supplementary results: intentional binding

A linear mixed-effects model was fitted to predict intentional binding (TC) based on action condition (active vs. control), feedback precision, performance error (|dWT|), delay, and their interactions. The model explained a marginal R² = .25 and a conditional R² = .43.

The model exploration revealed a significant effect of condition (β = -124.8, SE = 17.31, 95% CI [-158.7, -90.8]; χ²(1) = 10.11, p = .001), indicating greater intentional binding in the active (mean = -117.2 ms, SD = 144.9) than in the control condition (mean = -100.2 ms, SD = 131.7). A significant effect of feedback precision was revealed (β = 22.91, SE = 17.11, 95% CI [-10.62, 56.45]; χ²(1) = 6.00, p = .014), indicating greater intentional binding in the 250-ms feedback precision condition (mean = -114.7 ms, SD = 125.5) than in the 750-ms feedback precision condition (mean = -102.8 ms, SD = 124.8). The effect of delay was also significant (χ²(2) = 2204.46, p < .001). Post-hoc analyses revealed that intentional binding for the 900-ms delay (mean = -269.0 ms, SD = 122.7) was significantly greater than for the 600-ms delay (mean = -97.1 ms, SD = 134.8; p < .001) and the 300-ms delay (mean = 41.4 ms, SD = 154.4; p < .001), and that binding for the 600-ms delay was significantly greater than for the 300-ms delay (p < .001).

The two-way interaction between condition and |dWT| was significant (β = -0.015, SE = 0.017, 95% CI [-0.048, 0.018]; χ²(1) = 8.38, p = .004), revealing a steeper slope of the effect of |dWT| on intentional binding in the control compared to the active condition. However, neither of the two slopes was significantly different from zero (active condition: χ²(1) = 0.5, p = .48; control condition: χ²(1) = 0.2, p = .65).

The two-way interaction between feedback precision and |dWT| was also significant (β = 0.010, SE = 0.017, 95% CI [-0.023, 0.044]; χ²(1) = 4.27, *p* = .039), revealing a steeper slope of the effect of |dWT| on intentional binding in the 250-ms feedback precision condition compared to the 750-ms feedback precision condition. Again, however, neither of the two simple slopes was significantly different from zero (250-ms feedback precision: χ²(1) = 2.62, *p* = .11; 750-ms feedback precision: χ²(1) = 0.32, *p* = .57).

The two-way interaction between condition and feedback precision was significant (β = -1.71, SE = 24.17, 95% CI [-49.07, 45.66]; χ²(1) = 10.90, *p* = .001). Post-hoc comparisons revealed greater intentional binding in the 250-ms feedback precision condition for the active condition (mean = -131.4 ms, SD = 145.8) than for the control condition (mean = -97.8 ms, SD = 140.0; *p* < .001), greater binding in the active 250-ms condition compared to the active 750-ms condition (mean = -103.3 ms, SD = 154.8; *p* = .0003), and greater binding in the active 250-ms condition compared to the control 750-ms condition (mean = -102.3 ms, SD = 132.9; *p* = .0005).

A significant two-way interaction between |dWT| and delay was also found (χ²(2) = 18.27, *p* < .001), showing a positive slope of the effect of |dWT| on intentional binding in the 900-ms delay condition and a negative slope in the 300-ms delay condition, although neither simple slope was significantly different from zero (300-ms delay: χ²(1) = 1.72, *p* = .19; 900-ms delay: χ²(1) = 0.92, *p* = .34).

The interaction between condition and delay was significant as well (χ²(2) = 512.16, *p* < .001), with all post-hoc comparisons being significant (smallest *p* = .001).

The remaining effects, including all higher-order interactions, were not significant (all *p*-values > .23). Finally, the interaction between |dWT|, condition, and feedback precision was not significant, β = –0.03 (SE = 0.02), 95% CI [–0.08, 0.02], χ²(1) = 0.004, *p* = .95. **In particular, the**

The results are reported in **Figure 5c and d** in the main manuscript.

## Supplementary analyses: the effect of feedback value on intentional binding

#### Methods

To assess the potential influence of feedback valence on intentional binding, we conducted exploratory mixed-effects models including feedback value as a predictor. Condition (Active/Control) was also entered as a fixed effect, and Participant as a random intercept.

#### Results

The analysis revealed a significant main effect of condition (β = 14.19, SE = 9.19, 95% CI [-3.83, 32.20]; χ²(1) = 7.07, p = .008), confirming greater intentional binding in the active (mean = –117.2 ms, SD = 144.9) than in the control condition (mean = –100.2 ms, SD = 131.7). However, the effect of feedback value was not significant (β = -8.25, SE = 9.23, 95% CI [-26.34, 9.83]; χ²(1) = 0.65, p = .419), nor was the interaction between condition and feedback value (β = 5.84, SE = 12.90, 95% CI [-19.44, 31.11]; χ²(1) = 0.20, p = .651).

Experiment 4

#### Participants screening

As in Experiment 1, 2 and 3, we computed (for each participant) the rank correlation between trial number and |dWT|. A negative rho coefficient indicates a progressive reduction of adjustments in the motor plan throughout the task. Based on this index, we sought to exclude from analyses participants showing the inverse pattern. One participant (ID= 3) was excluded from analyses based on this criterion. See **Figure S4A**.

Similarly, to check for participants’ overall accuracy in the time estimation judgements, for each participant, we computed the rank correlation between the experimentally manipulated delay and the reported interval estimation. Based on this index, we sought to exclude from analyses participants showing a negative association between action-outcome delay and the estimated time interval. Five participants (ID=3, ID=4, ID=6, ID=10 and ID=13) were excluded from analyses based on this criterion. See **Figure S4B**.

**Figure S4: A.** Single-subject visualisation of the rank correlation between trial number and trial-to-trial changes in waiting times |dWT|. Green lines represent linear regression fits computed using least squares (ggplot2’s geom_smooth(method = "lm")). A negative slope indicates a progressive reduction in adjustments over trials, consistent with learning. **B**. Single-subject visualisation of the rank correlation between the experimentally manipulated action-outcome interval (ms) and the estimated action-outcome interval (ms).

## Supplementary analyses: Performance

#### Methods

#### The analyses for Experiment 4 exactly replicated those of Experiments 1, 2 and 3.

#### Performance

A linear mixed-effects model was fitted to predict log-transformed |Difference| values based on trial number, feedback precision, and their interaction. The model explained a marginal R² = .11 and a conditional R² = .19. The model exploration revealed a significant effect of trial number, β = –0.073 (SE = 0.004), 95% CI [–0.080, –0.066], χ²(1) = 817.25, *p* < .001, indicating greater |Difference| values for smaller trial numbers. A significant main effect of feedback precision was also observed, β = 0.114 (SE = 0.060), 95% CI [–0.005, 0.232], χ²(1) = 8.43, *p* = .004, suggesting greater |Difference| values (i.e., worse performance) in the 750ms feedback precision condition (M = 736.74 ms, SD = 1471.27) compared to the 250ms feedback precision condition (M = 700.69 ms, SD = 1411.25).
The two-way interaction between trial number and feedback precision was not significant, β = –0.002 (SE = 0.005), 95% CI [–0.012, 0.008], χ²(1) = 0.20, *p* = .66.

The results are reported in **Figure 7a** in the main manuscript.

## Supplementary analyses: Learning

#### Methods

#### The analyses for Experiment 4 exactly replicated those of Experiments 1, 2 and 3.

#### Results

A linear mixed-effects model was fitted to predict log-transformed |dWT| values based on trial number, feedback precision, and their interaction. The model explained a marginal R² = .05 and a conditional R² = .17. The model exploration revealed a significant effect of trial number, β = –0.055 (SE = 0.004), 95% CI [–0.063, –0.048], χ²(1) = 364.14, *p* < .001, indicating greater |dWT| values for smaller trial numbers. A significant main effect of feedback precision was also observed, β = –0.191 (SE = 0.067), 95% CI [–0.322, –0.059], χ²(1) = 22.43, *p* < .001, suggesting greater |dWT| values in the 250ms feedback precision condition (M = 724.75 ms, SD = 330.45) compared to the 750ms feedback precision condition (M = 673.76 ms, SD = 335.97).
The two-way interaction between trial number and feedback precision was not significant, β = 0.004 (SE = 0.006), 95% CI [–0.008, 0.015], χ²(1) = 0.39, *p* = .53.

The results are reported in **Figure 7b** in the main manuscript**.**

## Supplementary results: intentional binding

A linear mixed-effects model was fitted to predict intentional binding (TC) based on outcome relevance (instrumental vs. incidental), feedback precision, performance error (|dWT|), delay, and their interactions. The model explained a marginal R² of .39 and a conditional R² of .55.

The model exploration revealed a significant effect of outcome relevance, β = 0.77 (SE = 16.64), 95% CI [–31.84, 33.38], χ²(1) = 83.82, *p* < .001, indicating greater intentional binding in the Instrumental Outcome condition (M = –134.26 ms, SD = 125.67) compared to the Incidental Outcome condition (M = –88.38 ms, SD = 121.06).

The effect of delay was also significant, χ²(2) = 4932.49, *p* < .001.
Post-hoc analyses revealed that intentional binding for the 900 ms delay (M = –335.38 ms, SD = 142.3) was significantly greater than for the 600 ms delay (M = –113.97 ms, SD = 120.7, *p* < .001) and 300 ms delay (M = 116.68 ms, SD = 128.3, *p* < .001), and that binding for the 600 ms delay was significantly greater than the 300 ms delay (*p* < .001).

The two-way interaction between |dWT| and delay was significant, χ²(2) = 26.34, *p* < .001.

The interaction between Outcome Relevance and delay was significant, χ²(2) = 33.27, *p* < .001.

The three-way interaction between Outcome Relevance, |dWT|, and feedback precision was also significant, (β = 0.062 (SE = 0.029), 95% CI [0.005, 0.119], χ²(1) = 4.96, *p* = .026). The remaining effects were not significant (all *p* > .06).

The interaction was examined separately for each Outcome Relevance condition. One analysis assessed the effect of |dWT| and feedback precision in the Incidental Outcome condition, while the other focused on the Instrumental Outcome condition.

For the Incidental Outcome condition, we found a significant |dWT| × feedback precision interaction (Chisq(1) = 15.76, p < 0.0001), indicating opposite trends depending on feedback precision: a positive slope of |dWT| on intentional binding in the 750 ms feedback precision condition and a negative slope in the 250 ms feedback precision condition. However, the main effects of |dWT| (Chisq(1) = 2.08, p = 0.15) and feedback precision (Chisq(1) = 1.07, p = 0.30) were not statistically significant.

For the Incidental Outcome condition, we found a significant interaction between |dWT| and feedback precision (β = –0.0405, SE = 0.0102, 95% CI [–0.0605, –0.0205], χ²(1) = 15.76, p < 0.0001), indicating opposite trends depending on feedback precision: a positive slope of |dWT| on intentional binding in the 750 ms feedback precision condition (β = –0.0405, SE = 0.0102, 95% CI [–0.0605, –0.0205], p < 0.0001), and a non-significant positive trend in the 250 ms feedback precision condition (β = 0.0157, SE = 0.0079, 95% CI [0.0001, 0.0312], p = 0.049). However, the main effects of |dWT| (β = 0.0157, SE = 0.0079, 95% CI [0.0001, 0.0312], χ²(1) = 2.08, p = 0.15); and feedback precision (β = 18.38, SE = 11.88, 95% CI [–4.91, 41.67], χ²(1) = 1.07, p = 0.30) were not statistically significant. The marginal R² of the model was 0.005, and the conditional R² was 0.178.

For the Instrumental Outcome condition, no significant effects were observed (all p-values > 0.15).

Finally, we tested the slopes of the effect of |dWT| on intentional binding separately for each condition. The slope was significantly different from zero in the Incidental Outcome 750 ms feedback precision condition (β = –0.0257, SE = 0.0072, 95% CI [–0.0398, –0.0117], χ²(1) = 12.84, p = 0.0003), with a negative relationship between |dWT| and intentional binding. In contrast, the slope was also significantly different from zero in the Incidental Outcome 250 ms feedback precision condition (β = 0.0171, SE = 0.0080, 95% CI [0.0013, 0.0328], χ²(1) = 4.50, p = 0.034), showing a positive relationship between |dWT| and intentional binding.

The remaining post-hoc tests were not significant (all p-values > 0.57).

The results are reported in **Figure 7c and d** in the main manuscript.

## Supplementary analyses: the effect of feedback value on intentional binding

#### Methods

To assess the potential influence of feedback valence on intentional binding, we conducted exploratory mixed-effects models including feedback value as a predictor. Outcome Relevance (Instrumental/Incidental) and was also entered as a fixed effect, and Participant as a random intercept.

#### Results

The marginal R² of the model was 0.006, and the conditional R² was 0.160.

The analysis revealed a significant main effect of Outcome Relevance (β = –36.28, SE = 10.12, 95% CI [–56.12, –16.44], χ²(1) = .41.54, p < .0001), with greater intentional binding in the Instrumental Outcome condition (M = –134.26 ms, SD = 125.68) than in the Incidental Outcome condition (M = –88.38 ms, SD = 121.10). The effect of feedback value was not significant (β = 12.97, SE = 10.19, 95% CI [–7.00, 32.95], χ²(1) = 0.22, p = .64), nor was the interaction between Outcome Relevance and feedback value (β = –19.11, SE = 14.28, 95% CI [–47.11, 8.88], χ²(1) = 1.79, p = .18).
